# Supplementary material for: Trazodone, dibenzoylmethane and tauroursodeoxycholic acid do not prevent motor dysfunction and neurodegeneration in Marinesco-Sjögren syndrome mice
Source: PLoS One. 2025 Jan 13;20(1):e0317404. doi: 10.1371/journal.pone.0317404 (PMC11729928; doi:10.1371/journal.pone.0317404)
Supplement: Attachment — Submitted filename: 147177_1_rebuttal_3070929_sg099g.pdf [file pone.0317404.s002.pdf]

Full Revision

Review COMMONS

Manuscript number: RC-2024-02518
Corresponding author(s): Roberto Chiesa

1. General Statements [optional]

We much appreciated the reviewers' positive comments and constructive suggestions. We have made changes in the text and figures in accordance with their comments to address the issues raised, as explained in the point-by-point response below. In addition to the revised manuscript, we have provided an Excel spreadsheet containing, in separate sheets, the underlying numerical data for Figures (S1 data), and a version of the manuscript with track changes.

2. Point-by-point description of the revisions

Reviewer #1

This study provides negative in vivo evidence for the use of two PERK inhibitors and of TUDCA for the treatment of Sli1-related Marinesco-Sjögren syndrome (MSS).

Overall, the manuscript reports a substantial amount of work and the study could be published in its present format. The experiments are well described in terms of methodology and appropriate analysis has been applied. Claims are proportionate and not overstated

I would have only minor comments related to some clarifications that the authors could make in the present manuscript and a suggestion for experiments that could improve the manuscript.

First, although this is not my expertise, the in vitro analysis of CHOP luciferase assays suggests that very high concentrations, in particular of TUDCA, are needed to observe an effect. The authors may wish to clarify their opinion and whether this could be the reason why in vivo they have been unable to obtain any inhibition of the PERK pathway.

The reviewer is correct in pointing out that high concentrations of trazodone, DBM and TUDCA were required to inhibit the PERK pathway in the CHOP::luciferase reporter cell lines. However, as we state in the Discussion, we do not think that their lack of effect in vivo was due to insufficient drug levels, since woozy mice were treated with trazodone, DBM or TUDCA according to dose regimens and administration routes that have proved effective in other neurodegenerative disease mouse models. Moreover, our analysis did not find major differences in drug bioavailability between mice with the woozy genetic background (CXB5/ByJ)

and C57BL/6J mice in which these drugs had shown neuroprotective effects (see also the response to the next point).

Second, it seems to me that when measuring the Trazodone metabolism there is a difference between acute and chronic treatment. It would be worth discussing what the authors make of that and what is more relevant (I assume chronic) to the disease model outcome.

We realized that the nomenclature used in Figures 6 and 7 was confusing, leading the reader to think there were differences in trazodone levels between chronically and acutely treated mice.

The experiment shown in Figure 6 was designed to test whether there were differences in trazodone pharmacokinetics and metabolism between mice of the woozy strain, which have the CXB5/ByJ genetic background, and C57BL/6J mice in which trazodone had shown neuroprotective effects in previous studies. In contrast, Figure 7 illustrates the levels of trazodone and m-CPP in control and woozy mice (both of which have the CXB5/ByJ genetic background) that had been chronically treated with trazodone for 5 weeks. These are the same animals as in Figure 3, as we state in Figure 7 legend. Therefore one should compare the levels of trazodone and m-CPP in Figure 7 with those of the “woozy” group (CXB5/ByJ genetic background) in Figure 6. This comparison shows that trazodone and m-CPP levels are comparable after chronic and acute (6h) treatment.

To avoid confusion, we have changed the mouse nomenclature. We have renamed the control group of mice as “CT” (previously “WT”) throughout the text and figures. In Figure 6, we have used CXB5/ByJ instead of “woozy” to emphasize the comparison between the different genetic backgrounds (CXB5/ByJ vs C57BL/6J). Finally, we have replaced the colors of symbols in Figure 7 in order to match those of Figure 3. We have also made the description and discussion of these results clearer in the revised manuscript.

With respect to the experiments a simple and informative addition would be the evaluation of the PERK pathway in mice treated with TUDCA, as this is missing.

The effect of TUDCA treatment on the PERK pathway is shown in Figure 5, where we measured CHOP mRNA levels in Purkinje cells microdissected from mice treated with 0.4% TUDCA in the chow, and in Figure 9C and D, where we measured the percentage of CHOP-immunopositive Purkinje cells in the cerebellum of same groups of mice by immunohistochemistry.

Figure 10 illustrates the results of an additional experiment in which woozy mice were treated with 500 mg/kg TUDCA intraperitoneally (ip), to test whether this alternative dosing regimen was any better. Like the treatment per os, TUDCA ip had no beneficial effect on motor dysfunction. Therefore we deemed it unnecessary to check the effect on PERK pathway inhibition in this group of mice.

A more difficult but potentially more interesting line of investigation is that of searching for potential actions of Trazodone that are PERK independent and might be responsible for the partial rescue observed in the beam walking test, which is much more sensitive and specific than rotarod, so worth considering. Assuming authors want to go down this route and add significance to their study my suggestion would be an unbiased RNA seq from the brain samples they already have. However, this is a suggestion to steer the study towards a more positive outcome and it is not necessary to support their current conclusions.

We agree with the reviewer that it would be interesting to investigate the mechanism by which trazodone slightly ameliorated the motor performance of woozy mice in the beam walking test. In the Discussion, we speculated that this could be due to an effect of trazodone on cerebellar serotonergic neurotransmission, which would require electrophysiological investigations to demonstrate. Of course, other mechanisms may also be operative, which RNA seq may help identify, as the reviewer suggests. However, this would be a complex and lengthy investigation, the results of which would not change the main conclusions of the present paper. We plan to explore this line of investigation in a future study.

Reviewer #2

Lavigna et al. described the effect of Trazodone in Marinesco-Sjögren syndrome model mice. Although the results are somewhat disappointing, this research has provided fundamental evidence for the future development of MSS therapeutics. There are few minor comments to further improve the manuscript

Major comment
P14
"Trazodone metabolism to m-CPP was slightly impaired in woozy mice compared to C57BL/6J mice. This was evident from the m-CPP/trazodone ratio, calculated on the AUC0-t in the plasma, which was 0.34 in woozy and 0.67 in C57BL/6J mice."

Why was the concentration different between WT and woozy mice? Which organ mainly contributes to the metabolism of trazodone? Is the function of this target organ different between WT and woozy mice?
Similar to trazodone, m-CPP clearance from plasma was slightly faster in woozy than in C57BL/6J mice.
Is m-CPP eliminated via the kidney? Or liver? Why is there a difference? Does SIL1 functions in liver or kidney? Needs discussion. This is the same for brain m-CPP levels.

As explained in the response to the second comment of reviewer #1, “woozy” in Figure 6 referred to mice with the CXB5/ByJ genetic background, and in this experiment we compared

trazodone pharmacokinetics and metabolism between CXB5/ByJ and C57BL/6J mice. We have modified the nomenclature of Figure 6 and the Results to make this clear.

Trazodone undergoes extensive hepatic metabolism, and only a small percentage is excreted unchanged in the urine. Metabolism involves hydroxylation, oxidation and dealkylation reactions, forming in particular the 5HT-active metabolite m-CPP (by CYP3A4). This and other metabolites are mainly excreted in urine, as conjugates [1–3]. The slight differences in trazodone pharmacokinetics and metabolism between the CXB5/ByJ and C57BL6/J mice shown in Figure 6 is not attributable to loss of SIL1 function, since both groups of mice carried wild-type *Sil1* alleles, but is most likely due to subtle differences between the two strains, for example in the binding to plasma proteins, metabolic enzymes, transporters and/or the excretion processes. The available data do not allow to clarify this issue.

The main point, however, is that no major differences were found in the plasma and brain concentrations of trazodone between these two strains of mice, which could have explained the lack of efficacy of trazodone in woozy mice, as we now further stress in the revised Discussion.

Minor comments
P3 L5 mutation should be variant.

This has been changed.

P4 L1 eIF2a-P should be phosphorylated eIF2α (p-eIF2α). The reviewer prefers (p-eIF2α) than (eIF2α-p) throughout the manuscript.

There is no standard rule for indicating phosphorylated proteins, and phosphorylated eIF2α is referred to in various ways in different papers, with the "p" in capital or lowercase, preceding or following the protein name, separated by a dash or not. We would prefer to maintain the current nomenclature for consistency with our previous publications, unless the Editor deems otherwise.

P9 L11 M-CPP should be fully spelled out the first time it appears. m-Chlorophenylpiperazine (m-CPP)

M-CPP is spelled out the first time it appears in the Material and Methods, subheading Drug treatments and bioanalysis.

Please explain the difference between the expected function of trazodone and its metabolite m-CPP. Why m-CPP is not effective.

Based on the observation that mice of the woozy strain had lower brain levels of m-CPP than C57BL6/J mice (Figure 6), we hypothesized that the lack of effect of trazodone in woozy mice could be due to m-CPP mediating the PERK signaling inhibitory activity of trazodone. However,

Review COMMONS

experiments in CHOP::luciferase reporter cells demonstrated that m-CPP does not inhibit PERK signaling (Figure 2D). The precise mechanism by which trazodone inhibits PERK signaling is not known [4], which makes it difficult to speculate why its main metabolite, m-CPP, does not exhibit this activity.

P11 L3 Fig. 3 Fig. 3A and B?

Yes, we specifically refer to panels A and B of Figure 3. We have indicated this in the revised manuscript.

P11 L6 at 7 weeks of age?

We have re-done the statistical analysis by two-way ANOVA and reported the results in the legend to Figure 3. There is a significant difference between vehicle- and trazodone-treated woozy mice in the number of missteps when the two groups are compared globally. No statistically significant difference in the number of missteps is detected at specific time points by post-hoc analysis. There is no statistically significant difference between vehicle- and trazodone-treated woozy mice in the time to traverse the beam. The Results section has been revised accordingly.

P12 L17 ~4 times, 4 times? Please state the exact value.
Done.

Figure 7 Why are brain m-CPP levels higher than plasma levels? Is trazodone metabolized in brain tissue?
Trazodone is extensively metabolized in the liver through Cytochrome P450 (Rotzinger et al., 1999). It is well documented that m-CPP readily passes the blood-brain barrier, much better than the parent compound, explaining its high brain levels [2].

P19 L7 ISRIB; please fully spell out the first time it appears.

Done.

<u>References</u>

1. Rotzinger S, Bourin M, Akimoto Y, Coutts RT, Baker GB (1999) Metabolism of some “second”- and “fourth”-generation antidepressants: iprindole, viloxazine, bupropion, mianserin, maprotiline, trazodone, nefazodone, and venlafaxine. Cell Mol Neurobiol 19:427–442. https://doi.org/10.1023/a:1006953923305

2. Caccia S, Ballabio M, Samanin R, Zanini MG, Garattini S (1981) (--)-m-Chlorophenyl-piperazine, a central 5-hydroxytryptamine agonist, is a metabolite of trazodone. J Pharm Pharmacol 33:477–478. https://doi.org/10.1111/j.2042-7158.1981.tb13841.x

3. DeVane CL, Boulton DW, Miller LF, Miller RL (1999) Pharmacokinetics of trazodone and its major metabolite m-chlorophenylpiperazine in plasma and brain of rats. Int J Neuropsychopharm 2:17–23. https://doi.org/10.1017/S1461145799001303

4. Halliday M, Radford H, Zents KAM, Molloy C, Moreno JA, Verity NC, Smith E, Ortori CA, Barrett DA, Bushell M, Mallucci GR (2017) Repurposed drugs targeting eIF2α-P-mediated translational repression prevent neurodegeneration in mice. Brain 140:1768–1783. https://doi.org/10.1093/brain/awx074
