# Supplementary material for: Trazodone, dibenzoylmethane and tauroursodeoxycholic acid do not prevent motor dysfunction and neurodegeneration in Marinesco-Sjögren syndrome mice
Source: PLoS One. 2025 Jan 13;20(1):e0317404. doi: 10.1371/journal.pone.0317404 (PMC11729928; doi:10.1371/journal.pone.0317404)
Supplement: Attachment — Submitted filename: Comment for Editor Manuscript PONE-D-24-30565.docx [file pone.0317404.s003.docx]

**Manuscript Number: PONE-D-24-30565**

***Title:*** *Trazodone, dibenzoylmethane, and tauroursodeoxycholic acid do not prevent motor dysfunction and neurodegeneration in Marinesco-Sjögren syndrome mice*

**Dear Editor,**

I appreciate the opportunity to review this manuscript. Thank you for entrusting me with this task. Below are my comments for the authors to consider:

**Accept with Minor Revisions:**

The manuscript addresses an important topic and shows promise. The suggestions outlined above are minor but necessary to enhance clarity. If these revisions are adequately addressed, I recommend acceptance with minor revisions. This study offers valuable insights into the challenges of repurposing drugs for rare genetic diseases and could significantly contribute to the field.

Best regards,
